# Relationship Between Thyroid Hormone Levels and Mean Platelet Count and Volume: Quantitative Assessment

**DOI:** 10.7759/cureus.3421

**Published:** 2018-10-08

**Authors:** Sardar H Ijaz, Shakeel M Jamal, Rehan Qayyum

**Affiliations:** 1 Internal Medicine, Oklahoma University of Health Sciences, Oklahoma City, USA; 2 Internal Medicine, Central Michigan University College of Medicine, Saginaw, USA; 3 Internal Medicine, Virginia Commonwealth University, Richmond, USA

**Keywords:** mean platelet volume, platelet count, thyroid hormone

## Abstract

Background

The hormones of the hypophysis-thyroid axis (HTA), thyroid stimulating hormone (TSH), and thyroid hormones, L-thyroxine (T4) and 3, 3′, 5-L-triiodothyronine (T3), modulate the metabolism, differentiation, and proliferation of almost every cell in the body. Several studies have examined the effect of HTA hormones on platelet count and mean platelet volume (MPV), but have reported inconsistent results. Our aim was to examine the association between HTA hormones and platelet count and MPV in a large cohort of the adult population of the United States.

Methods

We used the continuous National Health and Nutritional Examination Survey (NHANES) which made available data on HTA hormones (1999-2000, 2001-2002, 2007-2008, 2009-2010, and 2011-2012) to examine the association between HTA hormones and platelet count and MPV. Analyses were performed with adjustments for the complex survey sampling methods of NHANES data. Unadjusted and adjusted generalized linear regressions were performed to examine the relationship between HTA hormones and platelet count and MPV. Regression models were adjusted for age, sex, race, alcohol use, smoking status, serum c-reactive protein, red blood cell folate, diabetes mellitus, glomerular filtration rate, body mass index, and hypertension.

Results

Of the 10,619 individuals eligible for inclusion in the analyses, 5,267 (49.6%) were females and 2,132 (20.08%) were African Americans. The mean ± standard deviation of platelet count was 256.4 ± 67.1 10^9^/L, MPV 8.04 ± 0.92 fL, serum T4 7.92 ± 1.68 mg/dL, and serum T3 114.08 ± 24.6 ng/dL. In unadjusted analyses, an increase in the serum levels of T4 or T3 was associated with a significant increase in the platelet count and MPV (all *p*-values < 0.05). In contrast, an increase in serum TSH level was associated with a significant decrease in the platelet count (*p*-value = 0.05) but had no effect on MPV. After adjustment for potential confounders, serum T4 levels were significantly associated with platelet count but not with MPV. Individuals in the lowest quartile of T4 had 18.73 x 10^9^/L lower platelet count than individuals in the upper-most quartile (*p*-value = 0.03). Serum TSH and serum T3 levels had no effect on platelet count or MPV after adjusting for potential confounding variables.

Conclusions

We report that only serum T4 levels, and not TSH or T3 levels, are independently associated with platelet count and there is no independent association between HTA hormones and MPV. Our findings suggest a possible role of serum T4 on thrombopoiesis or on platelet lifespan.

## Introduction

Platelets play an essential role in maintaining hemostasis and the vascular endothelium and in wound healing [1]. Platelet production or thrombopoiesis is a unique process in which a small number of megakaryocytes within bone marrow produce a large number of circulating platelets. A fine balance between thrombopoiesis and platelet apoptosis maintains the platelet count in blood, and various endogenous and exogenous factors can alter this balance [2-3].

Thyroid hormones, L-thyroxine (T4) and 3, 3′, 5-L-triiodothyronine (T3), modulate the metabolism, differentiation, and proliferation of almost every cell in the body [4]. Their production is regulated through a negative feedback mechanism by the hypothalamus-pituitary axis via thyroid stimulating hormone (TSH) [5]. Thyroid hormones mainly act via intranuclear thyroid receptors (TR), which combine with retinoid X receptors (RXR) to form a heterodimer. This heterodimer interacts with the thyroid response elements (TRE)-specific regulatory regions of genes in deoxyribonucleic acid (DNA) to modulate gene expression [6]. 

Thyroid hormone receptors are present on hematopoietic stem cells and serum levels of thyroid hormone may modulate the production of blood cells including platelets [7]. However, clinical studies have shown inconsistent associations between thyroid hormone levels and platelet count and mean platelet volume (MPV) [8-11]. Most of these studies were small and enrolled patients with clinical thyroid disease. Therefore, our objective was to examine the relationship between serum thyroid hormone levels (T4, T3, and TSH) and platelet count and MPV in a large cohort of adults without the history of thyroid disease.

## Materials and methods

Study population 

We used the continuous National Health and Nutritional Examination Survey (NHANES) to examine the association between thyroid hormones status and platelet count and MPV. The detailed methods for this survey are available online and briefly summarized here [12]. The continuous NHANES is a complex, stratified, multistage probability cluster conducted at regular intervals and is designed to be representative of the civilian, non-institutionalized US population. For each survey period, data are collected via household interviews and standardized physical examinations conducted in specially equipped mobile examination centers. 

We used those two-year survey periods for which data on thyroid hormone status were available (1999-2000, 2001-2002, 2007-2008, 2009-2010, and 2011-2012). Thyroid hormone levels were not measured during the 2003-2004 and 2005-2006 survey cycles. Participants who were older than 20 years, were free of clinical thyroid problems (answered ‘no’ to the question “ever told you had a thyroid problem/disease”) and had data on platelet count and thyroid hormones were included in the study. 

Study measurements

The demographic information was ascertained from self-reported responses to the questionnaire administered by trained interviewers. Body mass index (BMI) was calculated by dividing body weight in kilograms with height in meters. Blood pressure (BP) was calculated as an average of up to four readings. Hypertension was defined as a mean systolic BP of 140 mmHg or greater, a mean diastolic BP of 90 mmHg or greater, or a diagnosis of hypertension [13]. Based on alcohol intake, participants were categorized into non-drinkers, light drinkers (≤5 drinks/week), or heavy drinkers (>5 drinks/week). Participants who were active smokers were categorized as current smokers, and participants who had either quit smoking or had never smoked were categorized as non-current smokers. 

Blood counts, including platelet counts, were measured using Beckman Coulter Counter (Beckman Coulter Inc., Brea, CA, USA). Serum TSH was measured using the Access HYPERsensitive human thyroid-stimulating hormone (hTSH) assay (Beckman Coulter Inc., Brea, CA, USA) which uses a two-site immunoenzymatic (“sandwich”) technique. Serum T3 and T4 were measured by a competitive binding immunoenzymatic technique using the Access Total T4 and the Access Total T3 assays (Beckman Coulter Inc., Brea, CA, USA). T4 measurements in 2011-2012 were adjusted to compare with the rest of the survey periods. Serum C-reactive protein (CRP) levels were quantified using latex-enhanced nephelometry and a Behring Nephelometer II Analyzer (Behring Diagnostics, Somerville, NJ, USA). Serum creatinine was measured using the Jaffe rate method (kinetic alkaline picrate). Glomerular filtration rate (GFR) was calculated using the Chronic Kidney Disease Epidemiology Collaboration (CKD-EPI) equation [14]. Urine iodine concentrations were determined by inductively coupled plasma dynamic reaction cell mass spectroscopy (ICP-DRC-MS), which uses a multi-element analytical technique. Serum glucose was measured using a hexokinase-mediated reaction and blood glucose level was measured using the Beckman Synchron LX20 test (Beckman Coulter, Brea, CA, USA) on refrigerated specimens. Glycohemoglobin was measured directly using boronate affinity high-performance liquid chromatography (HPLC). Diabetes mellitus was defined as a diagnosis of diabetes mellitus, a glycohemoglobin > 6.5%, a fasting blood glucose > 126 mg/dL, or a random blood glucose > 200 mg/dL [15]. Red blood cell (RBC) folate levels were measured using the Bio-Rad Quanta phase II competitive protein-binding measurement procedure (Bio-Rad Laboratories, Hercules, CA, USA) from 1999 to 2002, or using microbiological assays from 2007 to 2012. RBC folate levels were adjusted for change in the measurement methods [16]. 

As different measures of iron status were available for different individuals, we used an algorithmic approach and used the next step in the algorithm only when data were not available for that particular step. A participant was defined as iron deficient if the ratio of soluble transferrin receptor (sTfR) to log10 (ferritin) was more than 1.36 [17]. If no data were available on soluble transferrin receptor, then iron deficiency was defined by transferrin saturation < 15%, serum ferritin < 12 ug/mL, and serum protoporphyrin > 1.24 umol/L. If there was no data on serum ferritin, transferrin saturation, or serum protoporphyrin, then iron deficiency was defined as either a transferrin saturation < 15% and serum protoporphyrin > 1.24 umol/L, or serum ferritin < 12 ng/mL and serum protoporphyrin > 1.24 umol/L, or transferrin saturation < 15% and serum ferritin < 12 ug/mL [18]. Soluble transferrin receptor levels were measured using the Tina-quant sTfR assay (Roche Diagnostics, Mannheim, Germany), an automated homogeneous immunoturbidimetric assay (Hitachi 912 clinical analyzer, Roche Diagnostics, Indianapolis, IN, USA). Serum ferritin was measured using the Bio-Rad Laboratories' "QuantImune Ferritin IRMA" kit (BioRad Laboratories, Hercules, CA, USA), a single-incubation two-site immunoradiometric assay (IRMA), from 1999 to 2002, and using the Roche Tina-quant ferritin immunoturbidimetric assay on the Hitachi 912 clinical analyzer (Roche Diagnostics, Indianapolis, IN, USA) from 2007 to 2012. Ferritin values were adjusted for 1999-2000, 2001-2002, and 2007-2008 to match 2009-2010 and 2011-2012 measurements. Free erythrocyte protoporphyrin was quantitatively determined by molecular fluorometry using a spectrofluorometer. Transferrin saturation was calculated by dividing serum iron by total iron-binding capacity (TIBC). Iron and TIBC were measured on an Alpkem Flow Solutions IV (rapid-flow analysis) system. 

Statistical analyses

Analyses were performed with adjustments for the complex survey sampling methods of NHANES data. In continuous NHANES, primary sampling units represent variance (sampling units used to estimate sampling error) units. Participants were oversampled for certain population subgroups such as African and Mexican Americans to ensure the reliability and precision of health estimate indicators in these subgroups. The sampling weights, and where needed subsample weights, were assigned to each person, reflecting the adjustment for the unequal probability of selection, nonresponse, and adjustments to independent population controls. Sample weights were constructed with the rescaling of weights such that the sum of weights matched the survey population at the midpoint of each two-year survey period. For this study, sample weights were constructed for 10 years of combined survey data. All statistical analyses were performed in the R statistical language (version 3.1.0) using the survey package (version 3.29) to account for the complex survey design.

Data were summarized as the mean and standard deviation, median and interquartile range, or percentages as appropriate. Although we excluded individuals with the history of thyroid disease, individuals may have subclinical disease. Therefore, we excluded individuals with serum TSH less than 0.1 or greater than 4.5 when examining the relationship between serum TSH and platelet indices. For total and free T3 and T4, we excluded individuals who were at the extreme 1% of the respective distributions. To examine the relationship independent of distributional assumptions, we also examined the relationship between quartiles of thyroid hormones and platelet indices. The distributions of serum CRP and RBC folate were right-skewed and these variables were log-transformed before analyses. Quartile cut points for TSH, T4, and T3 were based on weighted distributions in the whole study sample. Unadjusted and adjusted generalized linear regression models were built to examine the relationship between thyroid hormones and platelet count and MPV. Regression models were adjusted for age, sex, race, the extent of alcohol use, smoking status, serum CRP, RBC folate, diabetes mellitus, GFR, BMI, and hypertension. Data on iron deficiency was available on 4,513 individuals and in that subset, we included the iron deficiency in the model to examine its effect. 

## Results

The data from the 10-year period (1999-2002 and 2007-2012) included 51,446 individuals. We excluded 23,442 individuals for age < 20 years, 2,398 individuals for personal history of thyroid problems, 14,987 individuals for missing at least one serum thyroid hormone level platelet count, or MPV. Data on serum free T4, T3, and free T3 was available for a six-year period only (2007-2012) enrolling 7,781 participants. Of the 10,619 individuals included in the analyses, 5,008 (48.8%) were females, 2,016 (19.6%) were African Americans, 2,231 (21.8%) were current smokers, and 820 (9.4%) were heavy drinkers. The mean ± standard deviation age of participants was 49.4 ± 17.7 years, platelet count 256.7 ± 67.2 109/L, MPV 8.04 ± 0.92 fL, serum total T4 7.9 ± 1.5 mg/dL, serum free T4 0.79 ± 0.12 ng/dL, serum total T3 113.4 ± 20.4 ng/dL, serum free T3 3.17 ± 0.34 pg/mL, and serum TSH levels 1.68 (0.87) mIU/mL (Table 1). When examined by quartiles of total T4 levels, there were more females, nonsmokers, hypertensives, and diabetics in the highest quartiles of T4 than in the lowest quartile (Table 2). Similarly, individuals in the highest total T4 quartile had a higher BMI, platelet count, and mean platelet volume. However, there was no difference in age, GFR, and RBC folate among total T4 quartiles (Table 1).

**Table 1:** Relationship between thyroid hormones and other variables BMI: body mass index; RBC: red blood cell; GFR: glomerular filtration rate; TSH: thyroid stimulating hormone; T4: L-thyroxine; T3: 3, 3′, 5-L-triiodothyronine; SD: standard deviation

VARIABLES	T4 Quartile 1	T4 Quartile 2	T4 Quartile 3	T4 Quartile 4	Total	P-value
Age in years, *n*(SD)	49.17 (17.45)	49.49 (17.43)	49.68 (17.52)	49.18 (18.28)	49.38 (17.67)	0.67
Age categories						
Elderly (≥60 years), *n*(SD)	742 (28.91)	776 (30.22)	837 (31.41)	789 (32.01)	3,144 (30.63)	0.009
Middle age (40–59 years), *n*(SD)	880 (34.28)	868 (33.8)	870 (32.65)	731 (29.66)	3,349 (32.63)	
Young (<40 years), *n*(SD)	945 (36.81)	924 (35.98)	958 (35.95)	945 (38.34)	3,772 (36.75)	
Female, *n*(SD)	1,002 (39.03)	1,218 (47.43)	1,335 (50.09)	1,453 (58.95)	5,008 (48.79)	<0.001
Race categories n(SD)						
White	1,343 (52.32)	1,212 (47.2)	1,172 (43.98)	967 (39.23)	4,694 (45.73)	<0.001
Black	558 (21.74)	493 (19.2)	506 (18.99)	459 (18.62)	2,016 (19.64)	
Hispanic	538 (20.96)	695 (27.06)	814 (30.54)	856 (34.73)	2,903 (28.28)	
Other	128 (4.99)	168 (6.54)	173 (6.49)	183 (7.42)	652 (6.35)	
Alcohol usage categories, *n*(SD)						
Heavy drinker	245 (11.03)	224 (10.19)	199 (8.88)	152 (7.33)	820 (9.39)	<0.001
Lightdrinker	1,750 (78.76)	1,659 (75.44)	1,679 (74.89)	1,512 (72.94)	6,600 (75.55)	
Non-Drinker	227 (10.22)	316 (14.37)	364 (16.24)	409 (19.73)	1,316 (15.06)	
Hypertension, *n*(SD)	907 (35.33)	881 (34.31)	968 (36.32)	965 (39.15)	3,721 (36.25)	0.003
Diabetes mellitus, *n*(SD)	251 (9.78)	286 (11.14)	354 (13.28)	393 (15.94)	1,284 (12.51)	<0.001
Smoker, *n*(SD)	642 (25.03)	540 (21.04)	575 (21.59)	474 (19.26)	2,231 (21.76)	<0.001
BMI (kg/m^2^), *n*(SD)	27.77 (5.84)	28.6 (6.31)	28.89 (6.47)	29.57 (7.2)	28.7 (6.5)	<0.001
RBC folate, *n*(SD)	1,024.5 (587.1)	1,026.7 (540.2)	1,016 (548.6)	998.7 (573.6)	1,016.6 (562.5)	0.28
GFR (ml/min), *n*(SD)	95.12 (22.71)	94.73 (22.43)	94.44 (23.61)	94.29 (26.59)	94.64 (23.86)	0.67
TSH (uIU/mL), *n*(SD)	1.74 (0.9)	1.69 (0.85)	1.67 (0.86)	1.61 (0.84)	1.68 (0.87)	<0.001
T4 (ug/mL), *n*(SD)	6.14 (0.54)	7.31 (0.26)	8.27 (0.3)	9.94 (0.92)	7.9 (1.49)	<0.001
Free T4 (ng/dL), *n*(SD)	0.72 (0.09)	0.77 (0.1)	0.82 (0.11)	0.86 (0.12)	0.79 (0.12)	<0.001
T3 (ng/dL), *n*(SD)	105.2 (17.4)	110.8 (18)	115.8 (19.2)	122.6 (22.9)	113.4 (20.4)	<0.001
Free T3 (pg/mL), *n*(SD)	3.1 (0.32)	3.14 (0.32)	3.2 (0.34)	3.24 (0.35)	3.17 (0.34)	<0.001
Platelet count (10^9^/L), *n*(SD)	250.1 (64.9)	253.9 (65.2)	257.9 (65.6)	265 (72.1)	256.7 (67.2)	<0.001
Mean platelet volume (10^-15^L) *n*(SD)	7.98 (0.89)	8.02 (0.94)	8.05 (0.93)	8.1 (0.91)	8.04 (0.92)	<0.001

**Table 2:** Relationship between thyroid hormone profile and platelet count and mean platelet volume TSH: thyroid stimulating hormone; T4: L-thyroxine; T3: 3, 3′, 5-L-triiodothyronine; CI: confidence interval; REF: reference quartile

	Platelet Count (10^3^ platelets/uL)				Mean Platelet Volume (attoliters)			
	Unadjusted		Adjusted		Unadjusted		Adjusted	
	Mean difference (95% CI)	*P*-value	Mean difference (95% CI)	*P*-value	Mean difference (95% CI)	*P*-value	Mean difference (95% CI)	*P*-value
TSH (uIU/mL)								
TSH	-3.35 (-5.45 to -1.26)	0.002	-2.09 (-4.29 to 0.11)	0.06	-3.48 (-35.69 to 28.72)	0.83	12.28 (-21.8 to 46.36)	0.47
TSH Q1	REF		REF		REF		REF	
TSH Q2	-6.31 (-12 to -0.62)	0.03	-4.97 (-11.05 to 1.11)	0.11	55.01 (-24.68 to 134.7)	0.17	92.21 (-1.2 to 185.62)	0.05
TSH Q3	-6.27 (-11.35 to -1.19)	0.02	-6.66 (-12.37 to -0.94)	0.02	99.53 (30.36 to 168.69)	0.005	103.72 (24.62 to 182.82)	0.01
TSH Q4	-8.09 (-13.6 to -2.59)	0.004	-5.83 (-10.92 to -0.73)	0.03	-5 (-84.23 to 74.24)	0.90	49.73 (-30.38 to 129.85)	0.22
Serum T4 (ug/mL)								
T4	4.09 (2.9 to 5.27)	<0.001	2.03 (0.7 to 3.36)	0.003	28.12 (9.67 to 46.56)	0.003	30.92 (6.44 to 55.39)	0.01
T4 Q1	REF		REF		REF		REF	
T4 Q2	4.6 (0.11 to 9.08)	0.04	2.07 (-2.53 to 6.67)	0.37	39.38 (-32.76 to 111.52)	0.28	34.31 (-57.51 to 126.14)	0.46
T4 Q3	9.88 (5 to 14.76)	<0.001	4.07 (-1.06 to 9.21)	0.12	47.76 (-34.24 to 129.75)	0.25	74.23 (-25.29 to 173.75)	0.14
T4 Q4	15.65 (10.5 to 20.79)	<0.001	7.83 (1.84 to 13.82)	0.01	102.38 (28.49 to 176.28)	0.007	109.64 (1.1 to 218.18)	0.05
Serum Free T4 (ng/dL)								
Free T4	-27.84 (-47.27 to -8.42)	0.006	-18.87 (-39.39 to 1.65)	0.07	544.08 (191.9 to 896.26)	0.003	534.48 (151.85 to 917.11)	0.007
Free T4 Q1	REF		REF		REF		REF	
Free T4 Q2	-5.79 (-10.64 to -0.94)	0.02	-4.53 (-9.97 to 0.92)	0.10	107.78 (22.24 to 193.32)	0.01	89.94 (-12.01 to 191.9)	0.08
Free T4 Q3	-17.5 (-24.58 to -10.42)	<0.001	-15.64 (-24.06 to -7.23)	<0.001	282.47 (144.1 to 420.84)	<0.001	236.22 (90.14 to 382.3)	0.002
Free T4 Q4	-7.17 (-12.85 to -1.49)	0.01	-4.95 (-11.13 to 1.24)	0.11	123.59 (29.05 to 218.12)	0.01	124.42 (24.06 to 224.78)	0.02
Serum T3 (ng/dL)								
T3	0.09 (-0.02 to 0.19)	0.09	-0.05 (-0.17 to 0.07)	0.38	2.85 (1.16 to 4.54)	0.001	2.66 (0.65 to 4.66)	0.01
T3 Q1	REF		REF		REF		REF	
T3 Q2	4.35 (-1.55 to 10.25)	0.14	-0.53 (-6.72 to 5.67)	0.86	125.58 (40.97 to 210.19)	0.004	123.63 (16.77 to 230.49)	0.02
T3 Q3	5.03 (-1.52 to 11.58)	0.13	0.46 (-6.7 to 7.62)	0.90	58.5 (-46.67 to 163.67)	0.27	68.68 (-40.84 to 178.19)	0.21
T3 Q4	4.33 (-2.74 to 11.39)	0.22	-3.16 (-10.89 to 4.56)	0.41	176.63 (71.4 to 281.86)	0.001	160.25 (36.39 to 284.11)	0.01
Serum Free T3 (pg/mL)								
Free T3	6.84 (0.8 to 12.88)	0.03	4.32 (-3.71 to 12.36)	0.28	61.5 (-55.48 to 178.48)	0.30	6.03 (-105.51 to 117.56)	0.91
Free T3 Q1	REF		REF		REF		REF	
Free T3 Q2	-0.16 (-6.76 to 6.44)	0.96	-1.35 (-9.03 to 6.33)	0.73	121.5 (37.24 to 205.75)	0.006	116.67 (-0.22 to 233.56)	0.05
Free T3 Q3	2.31 (-4.48 to 9.09)	0.50	1.88 (-5.98 to 9.73)	0.63	290.15 (139.43 to 440.87)	<0.001	118.17 (12.21 to 224.13)	0.03
Free T3 Q4	4.04 (-1.96 to 10.03)	0.18	0.79 (-7.07 to 8.66)	0.84	131.44 (39.53 to 223.35)	0.006	56 (-60.14 to 172.14)	0.34

Effect of thyroid hormones on platelet count

Serum TSH levels were inversely associated with platelet count; each one mIU/L was associated with a decrease in platelet count by 3,353 platelets/uL (95% confidence interval (CI) = 5,451-1,255; *P* = 0.002). Similarly, individuals in the highest quartile of TSH had a significantly lower platelet count than individuals in the lowest quartile (difference = 8,093 platelets/uL; 95% CI = 13,599-2,587; *P* = 0.004). On the other hand, each one ug/mL of total T4 was associated with an increase in 4,085 platelets/uL (95% CI = 2,898-5,272; *P* < 0.001). However, there was an inverse relationship between free T4 levels and platelet count; each one ng/mL increase in free T4 was associated with a decrease in 27,842 platelets/uL (95% CI = 8,418-47,265; *P* = 0.006). Individuals in the highest quartile of total T4 had a significantly lower platelet count than individuals in the lowest quartile (Table 2). In contrast to total and free T4 levels, serum total T3 levels were not associated with platelet count while free T3 levels were associated with a significant increase in platelet count. Furthermore, there was no difference in platelet count between the quartiles of total T3 or free T3 (Table 2).

After adjustment for potential confounders, the association of TSH levels with platelet count was no longer statistically significant. However, individuals in the highest quartile continued to have a significantly lower platelet count than those in the lowest quartile (5,826 platelets/uL; 95% CI = 10,918-733; *P* = 0.03). Adjustment for potential confounders did not change the statistically significant association of serum total T4 levels with platelet count and the highest total T4 quartile had a significantly higher platelet count than the lowest quartile. However, free T4, total T3, and free T3 were no longer associated with platelet count after accounting for the effect of confounders (Table 2). 

Effect of thyroid hormones on mean platelet volume

There was no statistically significant relationship between serum TSH levels and MPV and between TSH quartiles and MPV except between the lowest quartile and the second-highest quartile. This relationship between the second-highest quartile and platelet count remained significant even after adjusting for potential confounders suggesting a possible nonlinear relationship. However, the inclusion of a quadratic function in the regression model did not find a significant association between TSH and MPV suggesting that the nonlinear relationship has a more complex shape. In contrast, we found a significant association between both total and free serum T4 levels and MPV which remained significant after adjusting for potential confounders (31 attoliters per ug/dL; 95% CI = 6.4-55.4; *P* = 0.01 and 534 attoliters per ng/dL; 95% CI = 151.8-971.1; *P* = 0.007, respectively). Individuals in the highest quartiles of total T4 or free T4 had higher MPV than individuals in the lowest quartiles and this relationship remained significant after adjusting for confounders (mean difference = 109.6 attoliters; 95% CI = 1.1-218.2; *p* = 0.05 and 124.4 attoliters; 95% CI = 24.1-224.8; *p* = 0.02, respectively) (Table 2). Similarly, total T3 levels were significantly associated with an increase in MPV and this relationship remained significant after adjusting for confounders (2.7 attoliters per ng/dL; 95% CI = 0.65-4.66; *P* = 0.01). However, there was no significant linear association between free T3 levels and MPV although a more complex nonlinear relationship was evident from the comparison of quartiles (Table 2).

## Discussion

In this large sample of a representative noninstitutionalized US adult population without a history of thyroid problems, we found a linear relationship between serum total T4 and platelet indices; an increase in T4 was associated with a rise in platelet count and MPV. On the other hand, free T4 levels and total T3 levels were positively associated with MPV but not with platelet count. Serum TSH levels and free T3 were not independently associated with platelet count or with MPV. Our results show that the relationship between platelet indices and thyroid hormones vary depending on the hormone and whether it is binding in serum. 

Our results may appear surprising at first as the serum levels of hypophysis-thyroid axis (HTA) hormones are inter-related and serum levels of one hormone are predictive of serum levels of other hormones within HTA [5]. On the other hand, our findings highlight an interesting relationship between platelet lifespan and the circulation half-life of HTA hormones.

Platelets are anucleate cells and, once produced by megakaryocytes and released from bone marrow into circulation, have a lifespan of 7-10 days [2]. As thyroid hormones act in the nucleus by modulating gene expression, these hormones can only affect platelets indirectly through megakaryocytes. T4, the main hormone secreted by thyroid glands, has a half-life of approximately 5-9 days and its levels may more closely reflect the T4 concentration to which megakaryocytes are exposed at the time of the release of platelets into the circulation [19]. The release of T4 is under the influence of TSH produced by the pituitary gland. Once released in the circulation, T4 undergo deiodination in the peripheral tissues to form T3. Serum TSH and T3 have relatively short half-lives (75 minutes and 24 hours, respectively) and their levels may fluctuate widely through the day and under various conditions; hence, one determination of TSH or T3 levels may not be reflective of the true average serum levels present during the formation and lifespan of platelets [5,11]. 

Previous studies that have examined the effect of thyroid hormones on thrombopoiesis have given inconsistent results [11,20-24]. Studies have either found no relationship or have found an inverse association between thyroid hormone levels and platelet count or MPV [25]. In contrast, we found a robust positive association between serum T4 levels and platelet count and MPV which remained significant after adjusting for potential confounders. This discrepancy between our study results and previous studies may stem from the fact that we excluded individuals with a personal history of thyroid disease while prior studies enrolled mainly individuals with thyroid disease and that these studies were small and thus were likely to not have been powerful to detect the relationship.

The biological mechanism through which T4 can increase platelet count is unclear although it is possible that thyroid hormones will affect both platelet formation as well as prolong the survival of platelets. In one study, bone marrow aspirates from the sternum were obtained from 26 hyperthyroid individuals and 16 healthy individuals [26]. Individuals with hyperthyroidism had a significantly higher number of megakaryocytes in bone marrow aspirates as compared to healthy individuals. One possible mechanism through which thyroid hormones may increase the number of megakaryocytes may include modulation of bone marrow matrix proteins, such as fibronectin. Indeed, in several cell lines, thyroid hormones increase the expression of fibronectin gene. Moreover, individuals with hyperthyroidism have elevated blood levels of fibronectin [27-28]. Fibronectin appears to affect megakaryocyte maturation and thrombopoiesis through interaction with integrin α4β1 [29]. Apoptosis is the major mechanism through which platelets die and thyroid hormones have been shown to inhibit apoptosis in several cell lines [30].

A major strength of our study is a large sample size representative of the adult US population. In addition, all clinical and laboratory variables were collected in a standardized manner. The cross-sectional design of the study is its major limitation, which makes it difficult, if not impossible, to assign a causal relationship between platelet count and serum T4 levels. Further, a single measurement of thyroid hormones may not provide the true thyroid hormone status of an individual over a longer time-period. 

## Conclusions

In conclusion, our findings demonstrate that serum total T4 levels are independently associated with increased platelet count and MPV, free T4, and total T3 are independently associated with MPV only, while serum TSH and free T3 levels have no association with either platelet count or MPV. Our study is unable to provide a mechanistic explanation of the observed association. Molecular and animal studies are needed to explore the biological mechanism through which thyroid hormones modulate thrombopoiesis and platelet lifespan.
